# How deep to dig: effects of web-scraping search depth on hyperlink network analysis of environmental stewardship organizations

**DOI:** 10.1007/s41109-022-00472-0

**Published:** 2022-06-06

**Authors:** Jesse S. Sayles, Ryan P. Furey, Marilyn R. ten Brink

**Affiliations:** 1 Oak Ridge Institute for Science and Education (ORISE) Fellow Appointed with the U.S. Environmental Protection Agency, Office of Research and Development, Center for Environmental Management and Modelling, Atlantic Coastal Environmental Sciences Division, Narragansett, RI, USA.; 2 Oak Ridge Associated Universities (ORAU) Contracted to the U.S. Environmental Protection Agency, Office of Research and Development, Center for Environmental Management and Modelling, Atlantic Coastal Environmental Sciences Division, Narragansett, RI, USA.; 3 U.S. Environmental Protection Agency, Office of Research and Development, Center for Environmental Management and Modelling, Atlantic Coastal Environmental Sciences Division, Narragansett, RI, USA.

**Keywords:** Social network analysis, Hyperlink networks, Web-scraping, Environmental governance, Decision support tools, Environmental stewardship

## Abstract

Social network analysis (SNA) tools and concepts are essential for addressing many environmental management and sustainability issues. One method to gather SNA data is to scrape them from environmental organizations’ websites. Web-based research can provide important opportunities to understand environmental governance and policy networks while potentially reducing costs and time when compared to traditional survey and interview methods. A key parameter is ‘search depth,’ i.e., how many connected pages within a website to search for information. Existing research uses a variety of depths and no best practices exist, undermining research quality and case study comparability. We therefore analyze how search depth affects SNA data collection among environmental organizations, if results vary when organizations have different objectives, and how search depth affects social network structure. We find that scraping to a depth of three captures the majority of relevant network data regardless of an organization’s focus. Stakeholder identification (i.e., who is in the network) may require less scraping, but this might under-represent network structure (i.e., who is connected). We also discuss how scraping web-pages of local programs of larger organizations may lead to uncertain results and how our work can combine with mixed methods approaches.

## Introduction

Understanding how and why different groups are connected is critical for addressing many of society’s most challenging sustainability problems, which often involve coordination and cooperation among different places and management sectors (Bodin 2017; Clark and Harley 2020; DeFries and Nagendra 2017; Sayles et al. 2019). Examples include non-point source pollution and emission reductions (DeFries and Nagendra 2017) and coordinating among jurisdictions that fragment interconnected land-and-sea-scapes (Crowder et al. 2006; Pittman and Armitage 2017). In response, academics and practitioners increasingly look at environmental governance, stakeholder, and management networks, often using social network analysis (SNA) tools and concepts^1^ (Bodin and Crona 2009; Bodin and Prell 2011; Bodin 2017; Bodin et al. 2019; Sayles et al. 2019; Groce et al. 2019; Kluger et al. 2020).

Most SNA research focusing on the environment relies on traditional social science methods, such as surveys and interviews, to collect primary data about network relationships (Bodin and Prell 2011). Recent scholarship, however, has used online information to understand these networks and is motivated by both an interest in understanding online interactions as a phenomenon of study and for their methodological potential to provide faster and cheaper approaches to collect data (Park 2003; Park and Thelwall 2006; Kreakie et al. 2016; Hayes and Scott 2018).^2^ Organizations can have an online presence through social media and websites, two different online sources that likely represent different kinds of network relationships (Hayes and Scott 2018). When deriving network relationships from organizations’ websites, information is usually extracted from descriptions in the body of the text, and can include information about roles and functions, or from hyperlinks to another organization’s page.^3^

Previous work on hyperlink networks has established some important observations about how hyperlink data might be used. Several SNA studies have compared results from data collected using online approaches to those using traditional survey approaches. They found that the two approaches resulted in similar results when analyzing small, core networks of environmental organizations, e.g., 25–60 groups (Morgans et al. 2017; Yi and Scholz, 2016). With larger networks (e.g., > 100), however, there tends to be much less similarity (Hayes and Scott 2018; Morgans et al. 2017; Yi and Scholz, 2016). Online data are likely not replacements for survey data, though this may be context specific; however, online data can be a valuable supplement or scoping tool. For example, Hayes and Scott (2018) found that structural patterns from online networks could be used to calibrate simulations to “fill in the gaps” of missing survey data, a classic research problem that can significantly undermine network analysis because it is very vulnerable to missing data (Costenbader and Valente 2003). Additionally, while online and survey derived data can result in different patterns of connection, several studies shows that there can be less variability about who simply is in the network (Kreakie et al. 2016; Morgans et al. 2017). This led Kreakie et al. (2016) to propose using online data as a tool to help identify stakeholders for collaborative environmental governance.

A key question when gathering data from websites is how deep to search. A website consists of any number of internally linked web-pages, collated under a unique Uniform Resource Locator (URL). The term ‘search depth’ refers to the minimum number of clicks that a user would navigate through to go from a website’s homepage to a sub-page within that website. Intuitively, searching more pages has the potential to uncover more network information, with a possible tradeoff of time and energy spent searching (whether it be computer automated or manually) versus the value of information returned for the purpose at hand. Among studies using automated web-crawlers and scrapers (terminology used synonymously here^4^), approaches have included searching an entire website (Ackland and O’Neil 2011), searching to depths two or three (Hayes and Scott 2018; Yi and Scholz 2016), searching a site’s home page (Elgin 2015), or the home page plus purposefully selected sub-pages titled “partners” and “links” found on the home page (Kreakie et al. 2016). Depth parameters for research using manual approaches to derive network data from website are rarely reported, though often cited as being systematic (Hileman and Lubell 2018; Hileman et al. 2018; Morgans et al. 2017; Berardo et al. 2019). In general, there has been surprisingly little discussion in the literature about best practices and tradeoffs associated with search depth, with a variety of depths used, often without explanation. A detailed assessment about how search depth affects SNA data collection and analysis would thus be useful for several reasons.

First, it is necessary to understanding how search depth affects network data collection and analysis to ensure that research results are robust and valid. Second, understanding the effects of search depth can enhance case study synthesis by specifying how different depth studies should be compared. It also highlights the need to have transparent and justified search depths reported in research papers. Third, understanding search depth has practical implications for stakeholders and practitioners looking to use web-based network tools. While it is true that gathering data online is often faster and cheaper than traditional survey or interview methods and can facilitate multiple time series of data collection (Kreakie et al. 2016; Hayes and Scott 2018); even with computer assistance, web-scraping can still take hours to days depending on the data set (Issuecrawler 2021). Given that many environmental practitioners find their time and resources stretched thin (Sayles and Baggio 2017a; Sayles 2018), improved guidance on how deep to search and potential tradeoffs is essential information for using web-scraping tools.

In this paper, we address the issue of search depth by analyzing hyperlink data scraped from the web-pages of 78 environmental stewardship organizations. We focus on hyperlink network data collection using an automated computer web-scraping tool, though our work is generalizable to manual search approaches as well. Specifically, we quantitatively assess how network structure changes with increased search depth by considering several metrics commonly used in SNA to describe network structure and function (Table 1; Bodin et al. 2006; Carlsson and Sandström 2008). We also analyze the depth at which specific sub-pages describing likely partnership or other inter-organizational relationships occur, to provide guidance on gathering online network data based on webpage names. Finally, we assess how the quantity of hyperlink returns varies by search depth and if there is any difference among stewardship groups with different primary foci to understand if our results are contextual to a specific kind of stewardship group or activity.

## Methods

### Organizational website selection

**Fig. 1:**
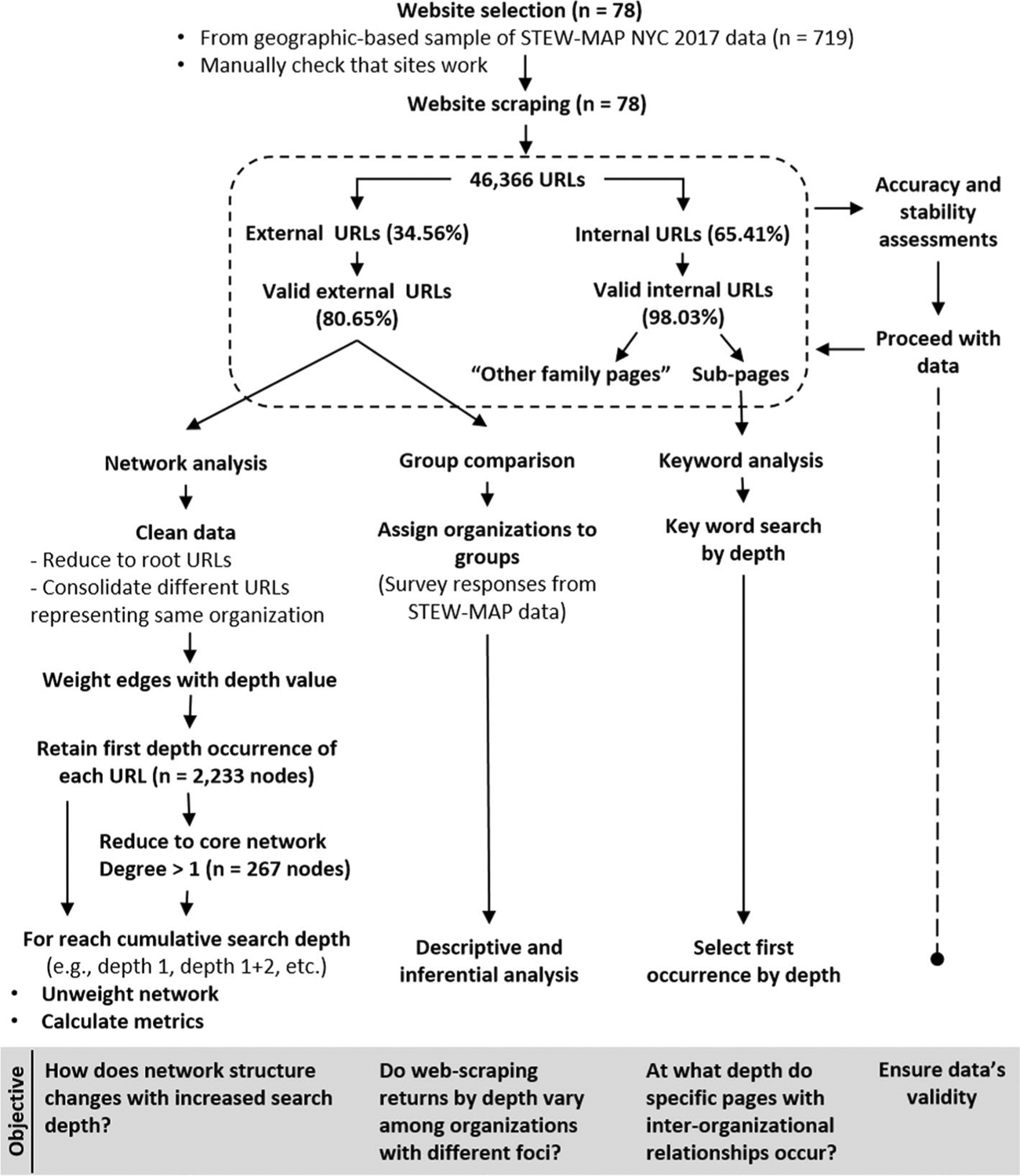
Summary of workflow and data preparation. External links connect to web pages with a different root URL than the searched site, while internal links connect to web pages with same root URL. Valid returns are working websites with a URL status code 200. Internal pages can be family or sub-pages, which is relative to the search URL as explained in the methods

We used data from the 2017 NYC Stewardship Mapping and Assessment Project (STEW-MAP) (USDA 2017), a relatively recent, publicly available data set about environmental stewardship organizations working in New York City, USA (n = 719). Since hyperlink web-scraping can be computationally slow (Issuecrawler 2021), we took a geographically bounded subset of the data for analysis selecting organizations that worked entirely within or overlapped the NYC borough of Staten Island (n = 111). The STEW-MAP data included organizations’ websites, which we verified resulted in 86 working websites; however, eight sites could not be scraped, which we removed from our final sample (n = 78, Fig. 1). See Additional file 1 for details.

### Web-scraping

We used the snaWeb package (version 1.0.1, Stockton 2020) in the R computational language environment (R Core Team 2020) to collect hyperlink network data. The snaWeb package is a web-scraper with a set of functions to retrieve URLs from specified websites and build hyperlink networks. snaWeb scrapes sites to any specified depth, checks the status of site URLs (e.g., URL status code 200 vs. 404 or other errors), and returns a redirected URL if one exists. The ability to find redirects is an important behavior for network studies, as two sites with hyperlinks to a common third site will be connected to this third site even if one site uses an outdated URL, which is a frequent issue on the web (Dellavalle et al. 2003; Duda and Camp 2008; Hennessey and Ge 2013; Jones et al. 2016; Hondula 2020).

We scraped the 78 websites between 09 and 17 June 2020 to a maximum search depth of ten, expecting most, if not all sites, to have a maximum depth below ten (see Additional file 1 for additional specification). Scraping returned 46,366 URLs; one third (34.56%) were external links to other sites; two-thirds (65.41%) were internal (i.e., they had the same root as the searched site). Most URLs (91.33%) were classified as valid returns, meaning they responded successfully when accessed (URL status code 200). While the number of URL returns differed among these categories, there was no difference in qualitative patterns or statistical comparisons (see Additional file 1). We therefore focus on valid external returns in the main text when analyzing network structure and group comparisons, as valid external returns are most likely to be potential network relationships. We then use valid internal sub-page returns for keyword analysis because these sub-pages would list an organization’s collaborators (Fig. 1).

To fully understand what information is returned from the web-scraper, it is important to understand how it responds to long URLs. Many large environmental organizations, such as government agencies or large non-profits, consist of sub-programs that in many ways, function more like independent programs than a single entity (Sayles and Baggio 2017a; Sayles 2018; Newig et al. 2010). For the purpose of understanding environmental governance systems, it often makes sense to treat these sub-programs as different groups. For example, when looking at stakeholders in the Northeastern United States, it is logical to include the U.S. Environmental Protection Agency (EPA) Region One, which works in the region, but not EPA Region Ten, which operates on the other side of the continent. Both regions, however, have the same root URL (www.epa.gov). snaWeb uses the full URL that is entered for the search (e.g., www.epa.gov/aboutepa/epa-region-1-new-england) as the search base. Sub-pages of this base are classified as internal sub-pages and scraped. Pages at the same level or higher (e.g., www.epa.gov/aboutepa/epa-region-10-pacific-northwest, or simply www.epa.gov) are classified as family pages having the same root, so technically internal, but not sub-pages, and are not scraped. This search behavior attempts to more accurately represent the structure and reality of networked environmental governance. Eight organizations in our Staten Island data self-identified by sub-pages (I.e., they listed sub-pages when replying to the STEW-MAP survey).

### Accuracy and stability assessments

We ran several stability and accuracy assessments to ensure our data’s validity (Fig. 1). To test accuracy, we compared hyperlink returns at depth one from the snaWeb package to manual inspections of the HTML source code for 19 sites (ten randomly selected and nine purposefully selected; 24.36% of our sample). The data generated with snaWeb had near 100% accuracy (see Additional file 1 for details).

To test for stability, we repeatedly scraped 26 sites (20 randomly and six purposefully selected; 33.33% of our sample) three times to see if there were fluctuations in the search depth and number of URL returns. The maximum search depth achieved per site was consistent, with zero percent variability across all three test runs. The total number of returned URLs was also stable (mean and median variability of 1.48% and 0.00%, respectively), with some variance attributed to slow-loading or unresponsive internal sub-pages that would be scraped when they did respond to the HTTP call of snaWeb, but not when they were unresponsive. Overall, snaWeb produced accurate and stable results in what is itself a highly dynamic and variable environment of the world wide web. (See discussion of potential limitations in the Additional file 1.)

### Hyperlink data preparation and analysis

The analysis of hyperlink network data almost always involves a significant level of data cleaning, re-coding, and consolidation (Ackland 2010; Elgin 2015). We reduced the total hyperlink data (n = 46,366 URLs, Fig. 1) to root URLs, as has been done elsewhere (Elgin 2015). It would not make sense, for example, for one of our search links to have multiple network connections to an external site’s “home,” “about,” and “partners” pages. We further removed links to social media, which show different but complementary information from hyperlink networks (Hayes and Scott 2018), and removed links to images, file storage, web-services, audio files, and any other content that did not represent an organization, as well as news media, which illustrates information distribution, but not necessarily inter-organizational connections.

Following Kreakie et al. (2016), we manually checked all URLs and consolidated them when two different root URLs represented the same organization (Fig. 1). For example, an organization might have a dedicated website, with a unique URL, to communicate its environmental management plan. We did not further modify root URLs from potentially related units, such as two academic units within a university (e.g., www.gc.cuny.edu and www.guttman.cuny.edu). Such consolidations should be guided by case specific information and the research questions at hand (Elgin 2015). Using the root URLs as reported (with the aforementioned cleaning) is suitable for testing network structure against search depth since our questions and analyses are largely methodological; however, other tests might warrant further refinement of URLs (Elgin 2015).

Once cleaned, all edges were assigned a value corresponding to their search depth. We removed all duplicate edges, retaining only the first instance and depth value. This yielded a network of 2233 nodes (i.e., URLs), which we further reduced, by removing all nodes with a total degree of one, to form a core network of 267 nodes (which included five isolates). We then unweighted the network and calculated metrics (Table 1) for each cumulative search depth, where returns for a given depth include those before it (Fig. 1), using the R packages intergraph, sna, network and igraph (Bojanowski 2015; Butts 2008, 2020a, b; Csardi and Nepusz 2006).

### Key word analysis and group comparisons

To identify the depths of specific sub-pages describing likely partnership or other inter-organizational relationships, we performed key word searches (listed in the results) for the first occurrence by depth in valid internal URLs (Fig. 1). To test for differences in total returns and maximum search depth based on stewardship foci we separated the STEW-MAP sample into two groups based on organizations’ stated primary stewardship objectives in the STEW-MAP database: first, organizations focused on communication-based activities, i.e., education and advocacy (EA, n = 40); second, groups doing ‘on-the-ground’ or management activities, i.e., conservation, management, monitoring, participating and partnering in stewardship activities, and transforming the environment (CMMPPT, n = 35, Fig. 1). Groups were compared using Mann–Whitney U tests in the R computation language (R Core Team 2020). Three groups were coded as unknown (two did not provide information, a third listed “none of the above”) and were not statistically compared due to the small sample.

## Results

**Fig. 2:**
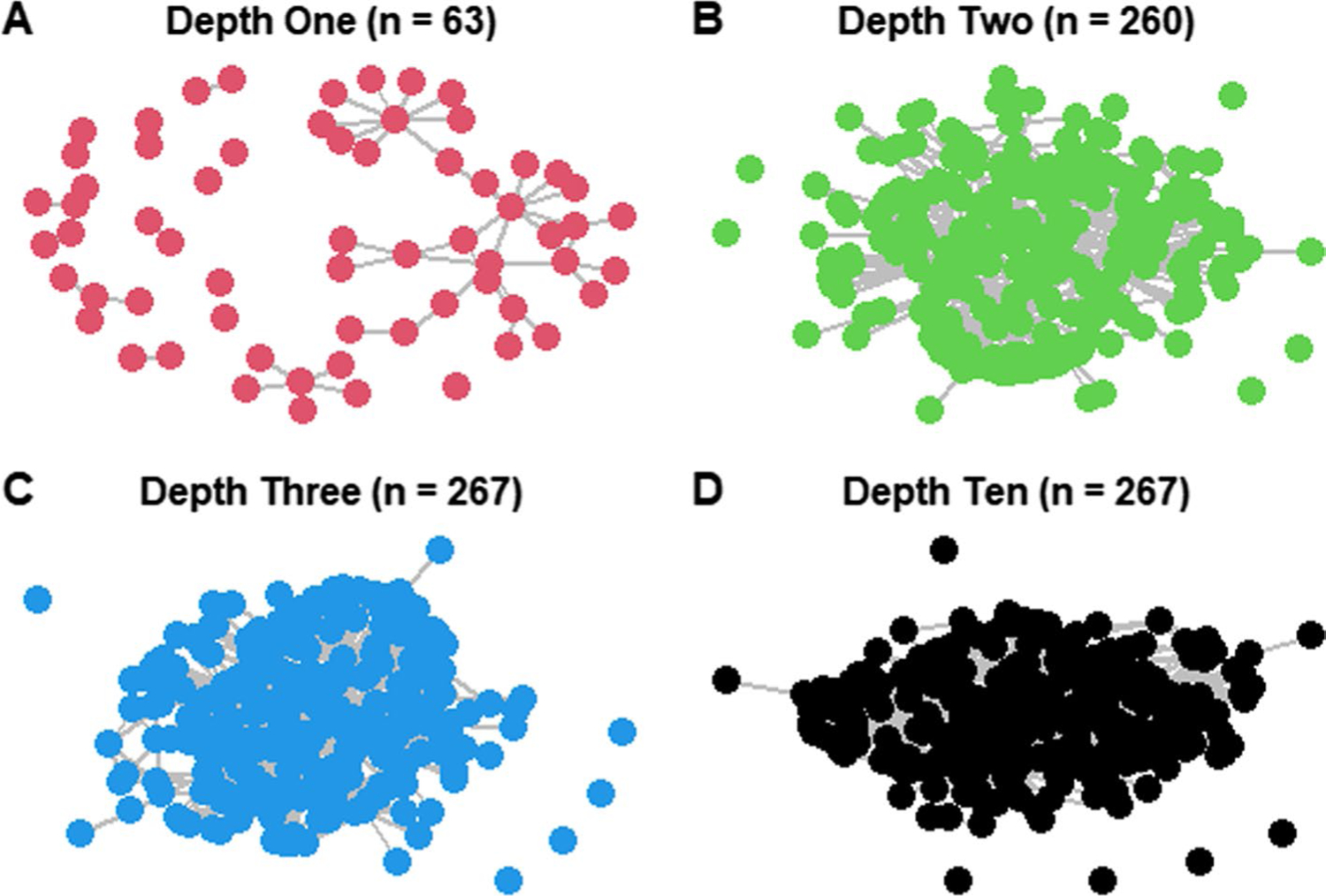
Network diagrams for the reduced root URL network at depths one, two, three, and ten, panels **A**–**D** respectively. New nodes were not added past depth three, thus depths four through nine are omitted for clarity and depth ten is shown for comparison. Nodes represent websites and edges represent hyperlinks from one site to another

**Fig. 3:**
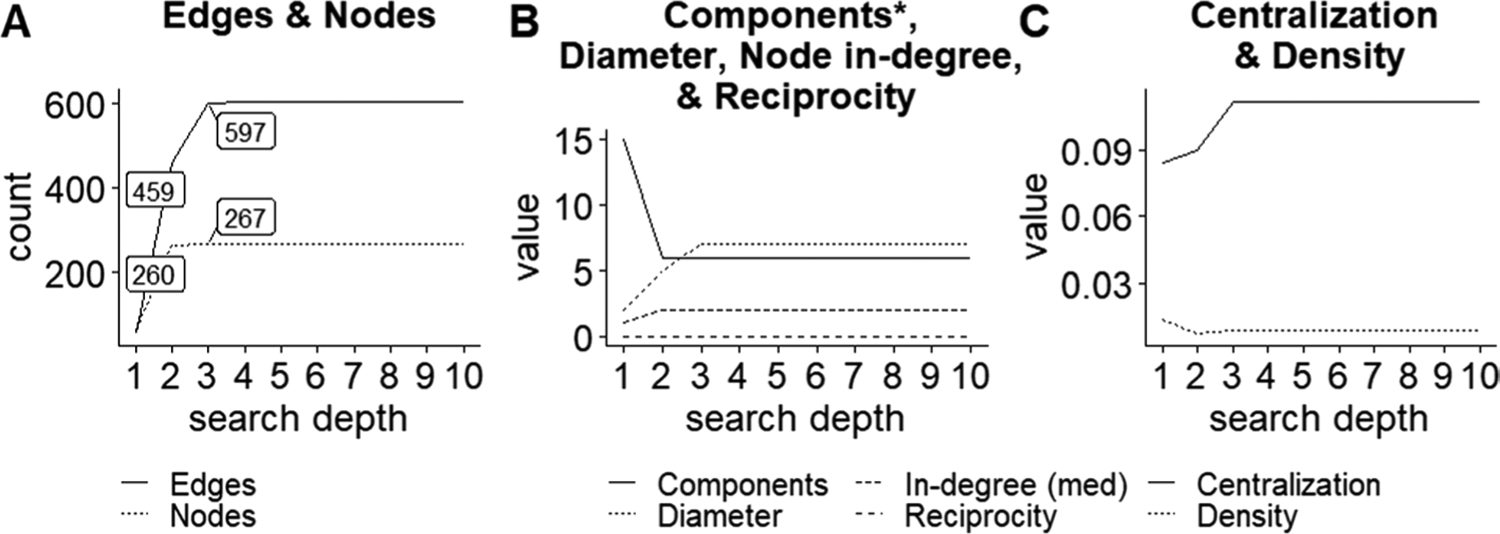
Panels **A**–**C** show node and graph level statistics for the reduced root URL network at different depths. Metrics are defined in Table 1. For reference, the number of edges and nodes returned at depths two and three are labeled in panel “**A**”, since these values are discussed in the text. Further values for all metrics are provided in Additional file 1: Table S1. (*The number of components does not include the 5 isolates in the graph, described in the methods)

The reduced root URL network grew rapidly from depth one to two and largely stabilized by depth three. There was little to no variability in calculated network metrics beyond depth three (Figs. 2, 3). Interestingly, while there was only a 2.70% increase in the number of nodes from depths two to three, there was a 30.07% increase in the number of edges (Fig. 3A, Additional file 1: Table S3). While network size (i.e., the nodes or URLs in the network) changed very little from depths two to three, the structure of who was connected changed dramatically. It is worth noting, however, that the non-reduced network (n = 2,333) did not show this pattern at depths two to three; nodes and edges were added at equivalent rates (22.77% and 27.12% respectively; Additional file 1: Table S3).

The first occurrences of within-URL key words meant to signifying inter-organizational relationship also occurred within the first three depths (98.4% of returns) and were concentrated around depths one and two (Table 2). Three key words, however, were only found at depths two or higher: “funding,” “donors,” and “team-members.”

**Fig. 4:**
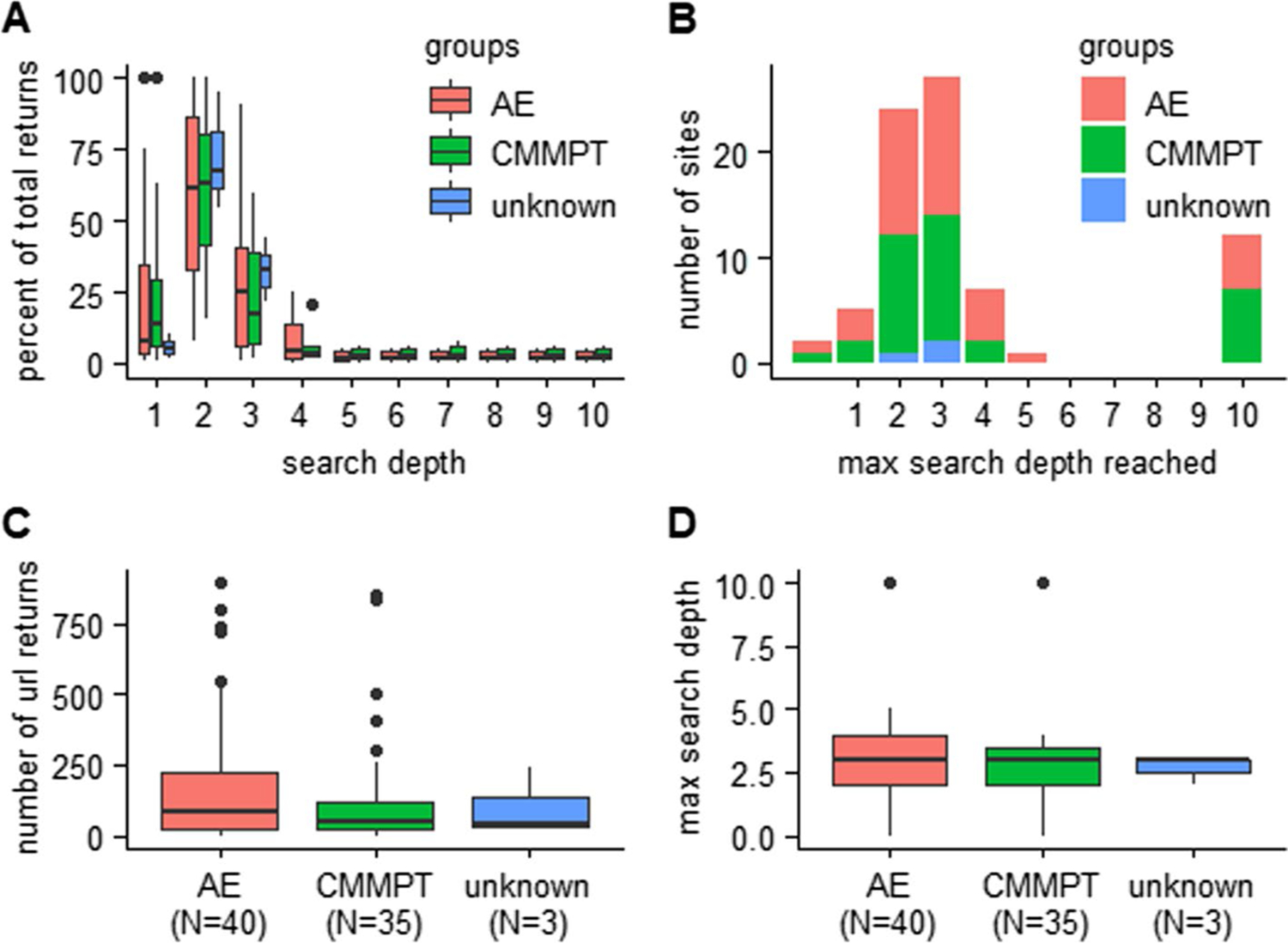
Boxplots for groups AE (advocacy and education focus), CMMPT (conservation, management, monitoring, partnership, and transformation focus), and unknown focus. **A** The percentage of a websites’ total valid external URL that were returned at each search depth; **B** the number of sites that achieved their maximum depth at a given depth; **C** the number of valid external URL returns for each group; and **D** the maximum search depth that the URLs were found. Stewardship focus was not reported for three organizations, which were classified as unknown. Groups AE and CMMPT were not statistically different (Mann–Whitney U test, **C** p = 0.375 and **D** 0.908). Unknowns were not statistically compared due to the small sample. Boxplots show the data’s distribution. The thick line in the middle of the box shows the median values, with the box itself bounding 25% of the data’s distribution above and below the median (i.e., the upper and lower quartile, respectively, which together make the interquartile range). Lines extend out to show the remaining data within the largest and smallest quarters of the datasets, but do not include extreme values, or outliers (defined as larger than 1.5 times the interquartile range), which are indicated as dots. If the median of two datasets falls within each other’s interquartile range, the distributions are generally not statistically different, which is confirmed by the Man-Whitney U tests for panels **C** and **D**

The raw scrape of the data also returned the majority of URLs by depth three or four (Fig. 4A), by which point, most sites reached their maximum depth (Fig. 4B). Even for 12 sites (15%) that reached the maximum search depth of ten (Fig. 4B), these higher search depths accounted for a very small percentage of their total URL returns (Fig. 4A) and there was little reward for the extra time needed to searching deeper.

Finally, the average number of valid external returns and maximum search depth were not statistically different between the AM and CMMPT groups (Mann–Whitney U test, p = 0.375 and 0.908, respectively, Fig. 4C, D); and all other comparisons were similar across the two groups (Fig. 4A, B).

## Discussion

A social network perspective is fundamental for addressing many environmental management and sustainability problems (Bodin 2017; Clark and Harley 2020; Sayles et al. 2019). Studying online presences of environmental organizations holds great potential to better understand environmental governance and policy (Hayes and Scott 2018; Yi and Scholz, 2016), as well as build tools to help environmental stakeholders and managers (Kreakie et al. 2016). Working with online network data is relatively new; we are still learning about best practices (Park and Thelwall 2006; Hayes and Scott 2018). This paper provides insight on a key variable for online research: how deep to search websites and whether this varies among organizations with different foci.

Based on our results, searching sites to depth three seems to capture all relevant network data. This does not vary among environmental stewardship organizations focused on communication versus on the ground management. Several of our tests suggest that simply searching to depth two could be appropriate in some cases. The majority of internal pages with possible relationships, based on key word search, occur within the first two depths; and the number of nodes (i.e., who was in the network) changes very little beyond depth two for the reduced focal network (n = 267). If simply scraping websites to identify major stakeholder groups, little information would be lost, in our case, by only searching to depth two. This could save environmental practitioners, some of whom lack time and resources (Sayles 2018; Sayles and Baggio 2017a), considerable computation time, data cleaning, and interpretation (Ackland 2010; Elgin 2015). However, scraping only to depth two would miss critical information about network structure (i.e., who is connected), as the number of edges did not stabilize until depth three, illustrating the importance of searching at this higher depth if one wants to analyze network patterns. Furthermore, for the full, unreduced network, many nodes were still added at depth three. While these are peripheral nodes within our network, they could be informative for certain investigations, such as identifying potentially marginalized groups. For structural analysis, searching to depth three, or maybe even four to be extra conservative, is likely best, unless other data justify something else. In the least, all search depth decisions should be clearly documented and reported in publications to improve cross-study comparisons and interpretations as search depth influences network structure.

While our case study clearly shows a sweet spot around depth three, regardless of an organization’s focus, several limitations are worth noting. First, our results need to be replicated for other locations and environmental issues beyond stewardship in order to build a stronger evidence base and set of guiding principles for online network data collections. Second, the sites in our sample predominantly represent non-profit and citizen organizations. Only two organizations represented other sectors: a sub-program within the NYC Department of Parks and Recreation and an academic unit within the City College of New York. It is possible that different organization types, such as state or federal government, may have different hyperlink patterns on their websites. While further testing is needed, we suspect that any differences among websites are more likely to be case specific as opposed to categorical. Nonetheless, our empirical results may be limited to non-profit and citizen groups. Third, results from the key word search reflect sites that use a description of the page in the URL, e.g., “our partners.” Analyzing cases where the page URL is not descriptive text, but rather a numeric ID or something else, could lead to different results; however, we are unaware of any theoretical reason why websites organized using numerical IDs in the URL would be different than those using text descriptions. These possible limitations noted, our results provide initial and important guidance on scraping websites to study online environmental organization networks.

In addition to our empirical results, working with the snaWeb tool revealed several interesting methodological issues relevant to the use and development of hyperlink network web scraping tools. For example, navigating the fuzzy boundaries that some organizations and sub-programs can exhibit is a known challenge when defining what a node represents in SNA studies about the environment (Sayles and Baggio 2017a; Sayles 2018; Newig et al. 2010). This problem may become more complex, however, when working with hyperlink networks as the existing fuzziness around defining proper units of analysis (i.e., what a node should represent) is compounded with website structure and all the choices that went into designing it. Several organizations in our sample self-identified by a sub-page of the root URL. When conducting research at a local scale, such as environmental stewardship activities in the Staten Island Borough of NYC, reducing the scrape of large Federal, State, and NGO websites to local programs or chapters makes a lot of sense (and indeed has been done elsewhere, e.g., Ackland and O’Neil (2011)). Yet, our own observations suggest, albeit anecdotally, that these sub-pages may not always match the actual organizational units engaged in collaborative environmental actions, which the network is meant to model. For example, some sub-pages had very few sub-pages of their own (“sub-sub-pages” if you will). These pages sometimes linked to what seemed like relevant internal organizational content stored elsewhere in the parent organization’s website. Any such content would not be scraped by the snaWeb package, or other similarly programmed web-scraper, because it is designed to only scrape sub-pages of the input search URL, including when that search URL is itself a sub-page of a larger website. These observations suggest that we need to better understand the relationships between sub-page scrape results and how these relate to the environmental management activities of sub-programs and chapters of larger organizations. Practitioners identifying stakeholders through web-scraping may want to be extra cautious when using sub-pages and integrate data from multiple sources and methods to ensure that they have not missed or over-represented specific stakeholders or sectors.

In general, mixed methods approaches may be fruitful for online network data gathering. Our research used an automated web scraping approach to gather hyperlinks, which is particularly attractive for its potential to gather data quickly, at low costs, and at multiple time series (Kreakie et al. 2016; Hayes and Scott 2018). This speed and potential to automate large datasets likely comes at the cost of more nuanced understandings about why relationships exist.^5^ In response, some researchers are employing qualitative manual coding of websites to be able to better discern what constitutes a network edge. For example, Hileman et al. (2018: 5) argue that for their study, “simply having a hyperlink or being mentioned on [a] website does not constitute a [network relationship]; partners [must] be clearly designated as collaborators on shared projects or other work activities.” Manual coding could make use of automated scraping to generate website attribute data, including the number of internal pages, external links to other sites, page names (which are extracted by the snaWeb package), URL key words, and other relevant information to guide more structured manual coding. Benefits might include more reproducible methods and enhanced case study comparisons. Such an approach is just one illustration about how mixed methods could be used. Future work should continue to build upon these methodological ideas.

Finally, organizations without a website cannot be documented by hyperlink web-scraping as there is no site to link. While not necessarily a limitation of our research, as our work focuses solely on the distribution of the hyperlinks that are present in a website, web-presence is a limitation of hyperlink web-scraping in general if the goal is to document and analyze environmental governance networks. Here again, the aforementioned mixed methods approaches can help. Manual coding, text mining, and machine learning might record organization names within website text that lack hyperlinks. We also observed in our work, however, that some organizations listed partners, funder, or other relations using images of logos without any hyperlinks. Manual coding and analysis may be needed in such cases.

## Conclusion

Network science tools and concepts are essential for addressing many environmental management and sustainability issues. Online network data provide important opportunities to understand environmental governance and policy networks, with potential cost and time savings compared to traditional research methods. Understanding how deep to search websites is important for building a scientific evidence base through comparable case studies and for developing efficient tools that can help stakeholders. Results from our analysis of the Staten Island, NYC data show that searching to depth three captures the majority of relevant network data and that organizations’ foci do not affect this. While searching to depth two may be sufficient for identifying key actors, it misses important structural information about who is connected. Future work should replicate our study for different places, environmental issues, and group types. We also need to better understand how to work with sub-programs of State and Federal agencies and NGOs. Researchers should also continue exploring creative methodological approaches such as combining automated methods to gather website metadata to inform systematic manual coding to better understand what links mean.

## Supplementary Material

Supplement1

## Figures and Tables

**Table 1:** Definition of several social network metrics that are commonly used in SNA studies about environmental issues and what the metrics imply for environmental governance and management and the use of online hyperlink data

Metric	Definition	Implications for environmental governance and management
Node count	The number of nodes in a network, which indicates network size.	Knowing the number of actors for a given environmental problem is a basic and important variable to ensure policies and solutions fit the situation at hand (Ostrom 2009). Implications are contextual to the specific issue or problem.
Edge count	The number of relationships among nodes in the network.	The number and distribution of edges in a network forms the foundation of a network perspective for environmental governance and sustainability (Bodin and Crona 2009; Bodin 2017). See the following definitions for implications.
Components	Subgroups within a network that are weakly connected or disconnected from each other. The number and size of components indicates how fragmented a network is.	Information and resources can travel faster in highly connected networks and poorly or not at all among fragmented components; however, hyper-connectivity can stifle innovation or foster the spread of undesirable information (Bodin et al. 2006; Vargas et al. 2020).
Median in-degree	Median number of incoming edges for a given node. In-degree assumes that edges have a direction, e.g., node A sends information to node B, as opposed to node A and B just sharing information with an undefined direction. In a hyperlink network, in-degree of node A is the number of hyperlinks going from other web-pages (i.e., other nodes) to node A.	Highly connected organizations can be influential and act as information or resource hubs; though maintaining many relationships can be taxing if lacking adequate resources (Bodin and Crona 2009). When hyperlinks represent positive affiliations among organizations (Hayes and Scott 2018) they can be interpreted as described above; however, hyperlinks might also represent negative motivations (Park and Thelwall 2006) and thus, interpretation of in-degree values can be contextual. These metrics also indicate network connectivity.
Network density	The proportion of total possible edges that exists in the network. Density ranges from 0 to 1, where 1 means all possible edges are present and 0 means none are present.	Higher density facilitates transmission of knowledge and resources, but can stifle innovation if ideas become homogeneous (Janssen et al. 2006). Dense networks tend to support cooperation and trust building (Berardo and Scholz 2010).
Network centralization	How edges in a network are distributed. Centralization ranges from 0, where all edges are distributed equally among the nodes, to 1, where a single node holds the network together.	High centralization can be efficient in settings with high levels of trust and agreement (Berardo and Scholz 2010; McAllister et al. 2017), but can also lead to, or result from, power imbalance in the absence of trust and agreement (Ernstson et al 2008; Bodin and Crona 2009). Structurally, centralized networks can be fragmented if central nodes are lost (Janssen et al 2006).
Graph diameter	The greatest distance (i.e., number of edges) between any pair of nodes. (For a disconnected network, diameter is calculated for the largest component).	Diameter indicates the potential distance that information or material might have to travel to get from one side of a network to another. All other variables being equal (e.g., levels of trust, shared objectives, etc.), shorter distances facilitate the flow of information and materials (McAllister et al. 2017).
Reciprocity	The percentage of edges that are reciprocated among two nodes; e.g., node A has a hyperlink to node B and B has a hyperlink back to A.	Reciprocity often indicates a stronger relationship. In collaborative environmental governance settings, reciprocity can reinforce trust building and reduce the risk of defection in high-risk collaborative processes (Berardo and Scholz 2010).

In online hyperlink networks, ‘nodes’ typically represent environmental organizations’ websites and edges represent a hyperlink from one website to another

**Table 2:** Counts of the first occurrences of key words describing inter-organizational relationship in valid internal sub-page URL strings by depth

Search term	Depth 1	Depth 2	Depth 3	Depth 4	…	Depth 8	Total
Collaborators	1	–	–	–		–	1
Contributors	–	–	–	–		–	0
Donors	–	1	1	–		–	2
Funders	3	1	–	–		–	4
Funding	–	3	4	–		–	7
Links	2	3	–	–		–	5
Members	11	5	5	–		–	21
Partners	14	7	1	–		–	22
Resources	17	5	–	–		–	22
Sponsors	4	2	–	–		1	7
Supporters	7	2	–	–		–	9
Team	18	3	–	1		–	22
Team-members	–	1	–	–		–	1
Total	77	33	11	1		1	123

Counts only include the first time a key word was returned in a URL per site to avoid biasing the data by repetition within a single site. No first returns were found at depths five, six, seven, nine, or ten, which are omitted from the table forclarity

## Data Availability

All analysis was done in the open source R computational language and relevant packages are cited in the main text. The data are publicly available at the U.S. Environmental Protection Agency’s (EPA) Science Hub repository (catalog.data.gov/dataset/epa-sciencehub) https://doi.org/10.23719/1522542. The datasets used and/or analyzed during the current study are also available from the corresponding author on reasonable request.

## Footnotes

1 Analyzing social network patterns can provide key diagnostics about how to enhance collaborative governance (e.g., Sayles and Baggio 2017a; Vance-Borland and Holley 2011), analyze issues such as power asymmetries or information flow (e.g., Bixler et al. 2016; Cohen et al. 2012), or test theories about building trust (e.g., Berardo and Scholz 2010; Lubell et al. 2014) and effective collaboration (e.g., Bodin et al. 2014; McAllister et al. 2017) to solve environmental problems. Network approaches to studying environmental management and sustainability are not limited to relationships among social actors. Network analysis can be used to understand more complex sets of relationships among organizations and policy forums (Lubell et al. 2014), organizations and environmental issues (Bergsten et al. 2019), and various kinds of social actors and the environmental resources that they interact with (Sayles et al. 2019; Kluger et al. 2020). These latter two examples are types of social-ecological networks (SENs; Sayles et al. 2019). There is, however, a core and foundational body of scholarship focusing on relationship among individuals or organizations involved in environmental governance, policy, and resource use (Bodin and Prell 2011).

2 The majority of scholarship using online data collection for environmental research focuses on social networks, where the network consists of organizations represented by their websites (Park 2003; Hayes and Scott 2018). Websites and social media, however, likely contain ample information about the places, issues, resources, and other policy and environmental information that could be used to analyze the more complex policy forum networks and SENs described in footnote one. (Indeed, such information has been gathered from websites to complement surveys and interviews for the analysis of a SEN (e.g., Sayles and Baggio 2017b)). Furthering our understanding about how best to use online information to study policy forum networks and SENs is likely a promising area for future research as traditional approaches to data collection remain a key hurdle to amassing empirical studies to support a larger evidence base (Sayles et al. 2019).

3 Websites might also be used to represent individuals, such as the home or bio-pages for prominent environmental thinkers, activists, or decision makers, but work to date has focused on organizations.

4 Web-crawlers are programs that automatically browse the internet and access pages by following hyperlinks. Scrapers are programs that extract specified content from web-pages. These processes can be done separately or simultaneously. When a web-crawler also extracts data during the crawl, it can be called a web-scraper (Khalil and Fakir 2017).

5 Machine learning and text mining may alleviate tradeoffs in depth vs. breadth. Further research on the effects of search depth on computation time and computer memory might also help understand and address technological limitations.

## Abbreviations

CMMPPT: Conservation, management, monitoring, participating and partnering in stewardship activities, and transforming the environment grouping
EA: Education and advocacy grouping
EPA: United States Environmental Protection Agency
NYC: New York City
SENs: Social-ecological networks
SNA: Social network analysis
STEW-MAP: Stewardship Mapping and Assessment Project
URL: Uniform Resource Locator
