# Supplementary material for: How deep to dig: effects of web-scraping search depth on hyperlink network analysis of environmental stewardship organizations
Source: Appl Netw Sci. Author manuscript; Available in PMC 2024 Jul 10. (PMC11235192; doi:10.1007/s41109-022-00472-0)
Supplement: Supplement1 [file NIHMS1816634-supplement-Supplement1.pdf]

Supplementary Materials

S.1. Supplemental data

Web scraping returns can be classified as external and internal, as explained in the main text. Sites can further be classified as valid (i.e., working websites) or not. In the main text we focused on valid external returns when analyzing network structure and group comparisons, as valid external returns are most likely to be potential network relationships. We then used valid internal sub-page returns for keyword analysis because these sub-pages would list an organization's collaborators. Figures S1 to S5 show data distributions for each category of site return, plus all combined. While the number of URL returns differed among these categories, there was no difference in qualitative patterns or statistical comparisons.

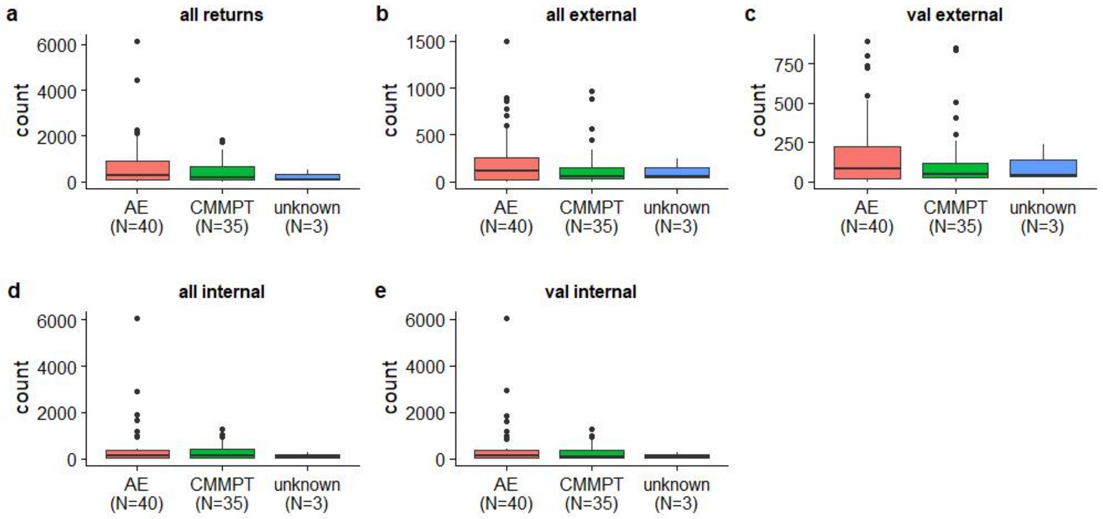

Figure S1. Total number of URL returns per group considering a) all returns, b) external returns, c) valid external returns only (status 200), d) internal returns, and e) valid internal returns only (status 200). There is no statistical difference between groups AE and CMMPT (Mann-Whitney U test, p = 0.429, 0.367, 0.375, 0.762, 0.766 respectively from a to e). Unknowns were not compared statistically due to the small sample. AE = stewardship foci advocate and educate. CMMPT = stewardship foci conserve, manage, monitor, partner, and transform. Panel c is reported in the main text. Boxplots show the data's distribution. The thick lines in the middle of the box show the median value, with the box itself bounding 25% of the data's distribution above and below the median (i.e., the upper and lower quartile, respectively, which together make the interquartile range). Lines extend out to show the remaining data within the largest and smallest quarters of the datasets, but do not include extreme values, or outliers (defined as larger than 1.5 times the interquartile range), which are indicated as dots. If the median of two datasets falls within each other's interquartile range, the distributions are generally not statistically different, which is confirmed by the Man-Whitney U tests.

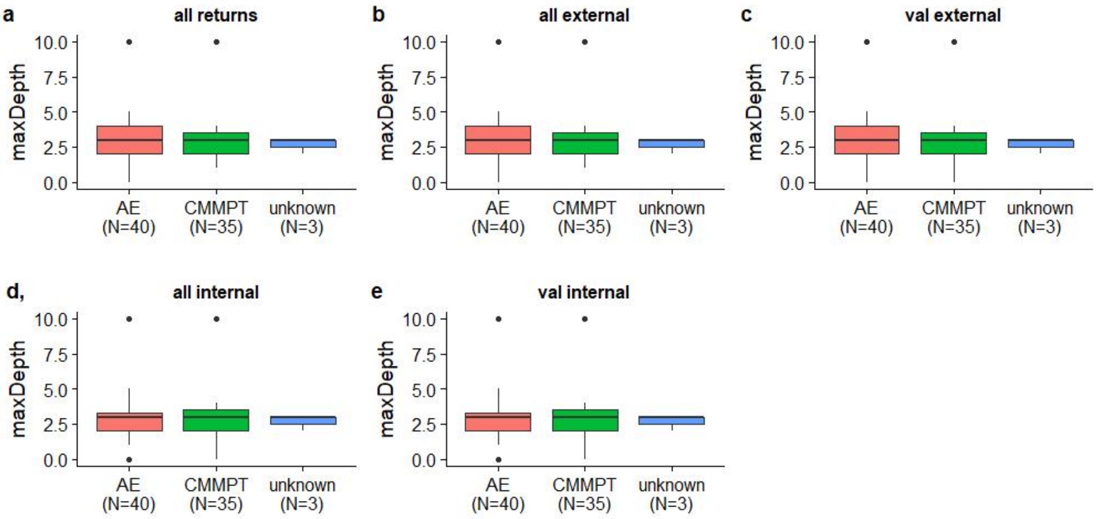

Figure S2. Maximum search depth reached per site among different groups considering a) all returns, b) external returns, c) valid external returns only (status 200), d) internal returns, and e) valid internal returns only (status 200). There are no statistical difference between groups AE and CMMPT (Mann-Whitney U test, p = 0.715, 0.890, 0.908, 0.485, 0.493 respectively from a to e). Unknowns were not compared statistically due to the small sample. See figure S1 for acronym definitions. Panel c is reported in the main text. See figure A1 for an explanation of boxplot interpretation.

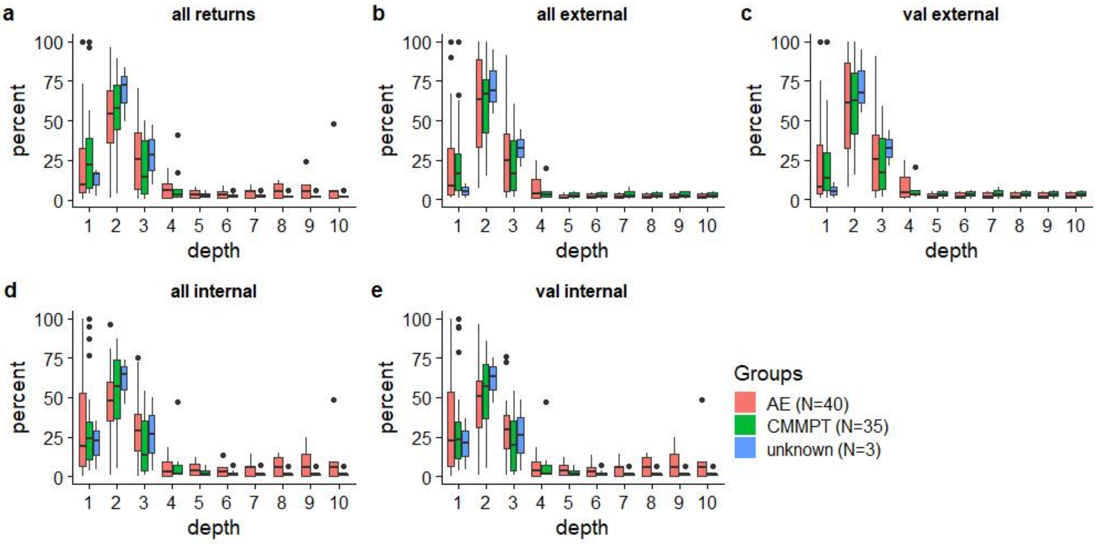

Figure S3. Boxplots of percentage of total returns per site at each depth per group considering a) all returns, b) external returns, c) valid external returns only (status 200), d) internal returns, and e) valid internal returns only (status 200). See figure A1 for acronym definitions. Panel c is reported in the main text. See figure S1 for an explanation of boxplot interpretation.

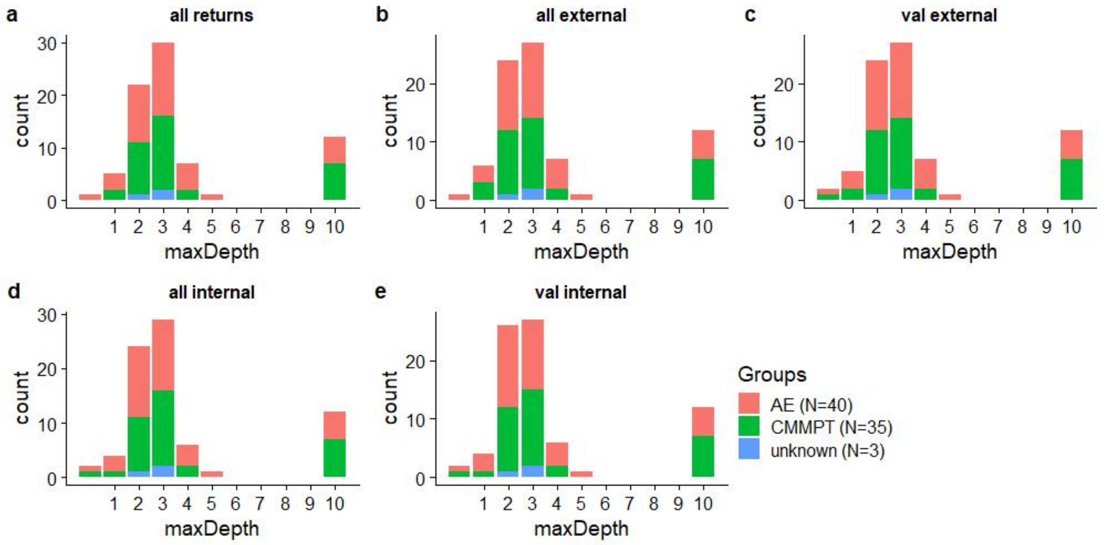

Figure S4. Number of sites that reach their maximum depth at a given depth per group considering a) all returns, b) external returns, c) valid external returns only (status 200), d) internal returns, and e) valid internal returns only (status 200). See figure S1 for acronym definitions. Panel c is reported in the main text.

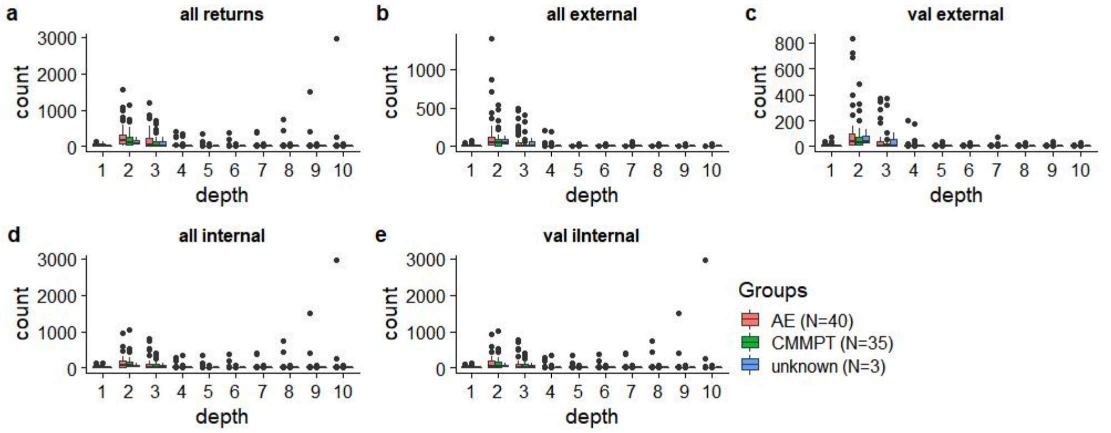

Figure S5. Total number of URL returns at each depth per group considering a) all returns, b) external returns, c) valid external returns only (status 200), d) internal returns, and e) valid internal returns only (status 200). See figure A1 for acronym definitions. Note, panel c is not reported in the main text as this is background descriptive information that does not directly address the research questions.

Figure S6 and tables S1 to S3 show additional data for the network analysis.

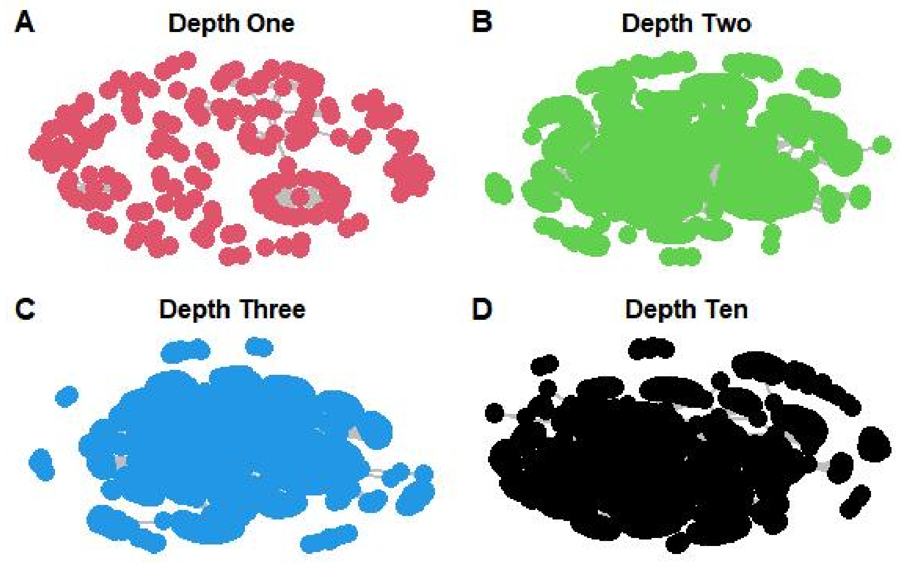

Figure S6. Network diagrams for complete, non-reduced, hyperlink network (n = 2,233) at depths A) one, B) two, C) three, and D) ten (the maximum depth searched). Circles represent nodes (i.e., websites) and lines represent edges (i.e., hyperlinks from one site to another). Network sizes and other statistics are reported in table S2.

Table S1. Node and graph level statistics for the reduced root URL network at different depths (reported in the main text and displayed in fig 3). Metrics are defined in table 1 in the main text.

Depth	Nodes	Edges	Comp-onents*	Average in degree	Median in degree	Graph density	Graph centralization	Graph diameter	Graph reciprocity
1	63	53	11	0.84	1	0.014	0.085	2	0.00
2	260	459	1	1.77	2	0.0068	0.090	5	0.0087
3	267	597	1	2.24	2	0.0084	0.11	7	0.010
4	267	602	1	2.25	2	0.0085	0.11	7	0.010
5	267	602	1	2.25	2	0.0085	0.11	7	0.010
6	267	602	1	2.25	2	0.0085	0.11	7	0.010
7	267	603	1	2.26	2	0.0085	0.11	7	0.010
8	267	603	1	2.26	2	0.0085	0.11	7	0.010
9	267	603	1	2.26	2	0.0085	0.11	7	0.010
10	267	603	1	2.26	2	0.0085	0.11	7	0.010

*the number of components does not include the 5 isolates in the graph

Table S2. Node and graph level statistics for the complete, non-reduced, hyperlink network (n = 2,238) at different depths. Metrics are defined in table 1 in the main text.

Depth	Nodes	Edges	Comp-onents	Average in degree	Median in degree	Graph density	Graph centralization	Graph diameter	Graph reciprocity
1	179	158	26	0.88	1	0.0050	0.029	2	0.00
2	1796	1990	11	1.11	1	0.00062	0.013	5	0.0020
3	2205	2531	10	1.15	1	0.00053	0.014	7	0.0024
4	2226	2557	10	1.15	1	0.00052	0.014	7	0.0023
5	2226	2557	10	1.15	1	0.00052	0.014	7	0.0023
6	2226	2557	10	1.15	1	0.00052	0.014	7	0.0023
7	2232	2564	10	1.15	1	0.00051	0.014	7	0.0023
8	2232	2564	10	1.15	1	0.00051	0.014	7	0.0023
9	2233	2565	10	1.15	1	0.00051	0.014	7	0.0023
10	2233	2565	10	1.15	1	0.00051	0.014	7	0.0023

Table S3. Percent change in nodes and edges among the reduced and full hyperlink networks for given depths. Changes beyond depth four are not reported due to their small size and because few changes were observed in the networks past depth three (tables S1 and S2).

**Change in depth**	**Reduced network (n = 270)**		**Full network (n = 2,238)**	
	**change in nodes**	**change in edges**	**change in nodes**	**change in edges**
1 to 2	312.70%	766.04%	903.35%	1159.49%
2 to 3	2.70%	30.07%	22.77%	27.12%
3 to 4	0.00%	0.84%	0.95%	1.03%

S.2. Details of organizational website selection

To select a geographically bounded subset of the 2017 NYC STEW-MAP data, we used the ‘select by location’ feature in ArcGIS version 10.6.1 to select all organizations that intersected (i.e., worked entirely within or overlapped) the NYC borough of Staten Island (n = 111). We applied a negative 250m buffer to omit organizations that would have only been included due to a small overlap, most likely due to inaccuracies in the spatial data.

We verified all websites, reported in our sample of the STEW-MAP data, using Google web searches and looked up alternative sites for non-working, redirected, or missing URLs. This resulted in 86 working websites; however, eight sites could not be scraped (see below), which we removed from our final sample (n = 78).

S.3. snaWeb package and search parameters

The snaWeb package's hyperlink scraping is carried out using the "buildNetwork" function (which calls upon several other functions within the package). We set the search to a maximum search depth of ten with a 180 second wait period for accessing sites before terminating the server call and registering a site as unresponsive. For all analyses, we used the build history resulting from buildNetwork, which is an output data frame reporting all information about the scrape including: all scraped URLs, their classification as internal, external, or sub-page (as described in the main text), scraping and access times, website page names, status code, and other information. In addition to the build history, buildNetwork also produces pre-formatted node and edge lists that can be used for network analysis and visualization. The contents of these pre-formatted lists can be specified to include internal, external, and sub-page sites. We used the build history for all analysis, however, as it contains more information than the pre-formatted node and edge lists and provided us more control over data selection, cleaning, and formatting.

S.4. Data scraping accuracy, stability assessments, and data integrity

We performed several tests to validate the snaWeb package's scrape results and ensure our data's integrity. To assess accuracy of the scrape results, we compared URLs returned by the snaWeb package with the manual inspection of the web-page source code for 24% of our sites: ten randomly and nine purposefully selected, which covered all sub-page URLs in our data, an item we specifically wanted to investigate (table S3). We scraped each site with the

snaWeb package at depth of one and compared the results to manual inspections of URLs in the webpages' source code. We copied each page's source code html into a document editor using Google Chrome Browser's "view page-source" function and inserted a paragraph break before instances of the text "href" in the code using "find and replace all." We then sorted the text to group all hrefs, copied these into a spreadsheet editor, and isolated the URLs from the wider line of html code using text to column functions and quotes as delimiters. We removed content that the snaWeb omitted by design such as website formatting content. We then manually compared the source code URLs to those returned by snaWeb using "if equals" logic statements to help us match text strings. In our comparisons we accounted for URL redirects, which snaWeb returns, and commented-out source code on the site, which snaWeb omits. (Commented-out source code is text that has been wrapped in special characters telling the computer to ignore it. This allows programmers to omit passages without deleting the code, or to include notes about the code. By omitting commented-out code, snaWeb only returns "live" content that would appear when viewing the website on the internet.) For all accuracy assessments, the scrape and manual source code inspection were done immediately after each other to control for time and potential website updates.

For the 19 sites, we had near 100% accuracy (table S3). There was only one case where the snaWeb package missed a URL for an unexplainable reason. The missed site was a "donate to us" link that redirected to a PayPal account, which may explain the omission. Testing also revealed a systematic omission when searching using a sub-page URL. The tool omitted the root URL in the build history, which is likely due to how the tool was programmed for handling sub-page as the search URL. This "root-dropping" is only an issue when entering a sub-page for the search. Root-dropping affected five of our searched sites as the root was not included in the source code for the other three sub-page searches. Since the

root-dropping is predictable and does not affect the overall search behaviour of the tool -- the dropped root would never itself be scraped for further URLs as it is not a sub-page of the original search -- the issue does not undermine the web-scraper's accuracy. Additionally, the five omitted sites account for 0.01% of the total number of URLs in our dataset resulting from the web-scraping and thus, including or omitting these five URLS would not change our results. Lastly, one site produced an unexpected return when it was scraped. Searching urbanjustice.org returned a link to the American Bar Association that was not present in the source code. Interestingly, the returned American Bar Association page had a link back to an urbanjustice.org program. We cannot explain this finding, though it could be linked to the server call and redirect behavior of the snaWeb package and illustrates that the World Wide Web is complex and dynamic. This extra return seems to be an isolated issue. No other assessed pages returned unexpected links.

To assess if the snaWeb package returned results consistently, we ran stability tests on 33% of our sites: 20 randomly and six purposefully selected sites to capture all sites that were only searched to a depth of one (table S4). We compared the maximum search depth achieved and the number of URLs in the build history. All tests for a given website were done using the same computer and internet connection and executed on the same day to control for time and possible website changes or update. Stability tests were not done on the same days as the original data scrape (i.e., scraping all 78 sites); therefore, there are some differences between the original data and the stability test.

The majority of the stability tests were stable (table S4). There were no fluctuations in search depth. Notwithstanding one site that had a large fluctuation (32.01%), variability in the build history was minor (min = 0%, max = 2.75%, mean = 1.48%, and median = 0%) resulting from non-responsive pages that would not be scraped if the snaWeb package was

unable to access them. Indeed, we observed that the most variable page, urbanjustice.org, was very slow to respond to Google Chrome Browser searches and likely suffered from many pages timing-out.

In addition to accuracy and stability, we investigated eleven sites that were only scraped to a depth of one (table S6). While stable (table S5), we wanted to confirm that this behavior was logical and that nothing was blocking a deeper scrape. We only investigate single depth returns as we felt that if the tool advanced to depth two, there was no logical reason why it would not advance to the maximum possible depth for a given site.

None of these cases resulted from a failed or flawed search. All resulted from logical behavior given how the snaWeb package performs web-scraping (table S6). In six cases there were no sub-pages to be returned and we retained these sites in our dataset. The other five sites did return sub-pages in the build history, but they were not recognized by snaWeb for logical reasons and reveal the web-scraper's limitations or sensitivities. We excluded these five cases from our data set.

One site had sub-pages, but all were returned with a 406 error indicating incompatibility between the client request and server response. Three sites had landing pages that redirected to some kind of sub-page. For example, the landing page for americanscottishfoundation.org redirects to americanscottishfoundation.com/home/index.html. All sub-pages of the site, however, come directly off of the root url and not "…/home/index." Therefore, the sub-pages are not recognized as sub-pages of the search URL. Lastly, two sites used a query operator (i.e., a question mark) in front of all sub-pages. Query operators in a URL instruct the server to take specific actions (such as look something up). This content appears to be ignored by the

snaWeb package to improve efficiency, for example, by not looking up hundreds of entries in a site's calendar. It does, however, reveal a limitation of the web-scraper, which cannot handle searching formats that employ query strings (e.g., .cfm format).

Lastly, two websites could not be scraped because they were blocked by a firewall and a third site caused the snaWeb tool to crash due to a problem parsing an xml document object. These three sites, plus the five sites that could not be scraped passed depth one, account for the eight omitted sites referred to in the main text.

Table S4. Accuracy assessments for 19 sites: 10 randomly and 9 purposefully selected, denoted with a "p" in column 1

number	name	Links on web page source code	Expected returns from snaWeb	Unexpected returns from snaWeb
1	urbanjustice.org	54	54	1
2	statenislandpps.org	44	44	0
3	billionoysterproject.org/	39	39	0
4	ny4p.org	40	40	0
5	anhd.org/	82	82	0
6	statenislandoutloud.org	22	22	0
7	shorewalkers.org	26	26	0
8	lincnyc.org	31	31	0
9	tibetanmuseum.org	43	43	0
10	peec.org	135	134	0
p1	americantowns.com/ny/statenisland/ organization/friends-of-abandoned -cemeteries-inc-s-i	97	97	0
p2	littleneck.net/lionsclub/index.htm	3	2	0
p3	greenmap.org/nyc	72	72	0
p4	nature.org/en-us/about-us/ where-we-work/united-states/new-york/	87	87	0
p5	streetlab.org/uni-project/	35	34	0
p6	nycgovparks.org/trees/tree-care/ ny-tree-trust	55	54	0
p7	scgis.org/chapters/pages/ new-york-and-new-jersey	53	52	0
p8	ssa.ccny.cuny.edu/programs-centers/ graduate-programs/m-land-arch-i/	107	106	0
p9	goingcoastal.org	0	0	0

Table S5. Stability tests for 26 sites: 20 randomly selected and 6 purposefully selected, denoted with a “p” in column 1. Percent variability (% var) indicates the variability between the minimum and maximum test run. Columns labeled “data” show values of depth and build for the sites when they were run as part of the total data-set. These numbers are not directly comparable to the stability tests because the stability tests were run several weeks after the total dataset and website content updates could have been made in that time. Note, long URLs have been truncated to improve legibility.

		Search depth					Build history				
number	name	Run 1	Run 2	Run 3	% var	data	Run 1	Run 2	Run 3	% var	data
1	urbanjustice.org	3	3	3	0.00	3	495	631	728	32.01	910
2	statenislandpps.org	3	3	3	0.00	3	153	153	153	0.00	154
3	billionoysterproject.org	3	3	3	0.00	3	411	411	411	0.00	403
4	ny4p.org	3	3	3	0.00	3	939	939	939	0.00	911
5	anhd.org	10	10	10	0.00	10	1874	1894	1906	1.68	1816
6	statenislandoutloud.org	3	3	3	0.00	3	98	98	98	0.00	98
7	shorewalkers.org	3	3	3	0.00	3	240	240	240	0.00	234
8	lincnyc.org	2	2	2	0.00	2	106	109	109	2.75	100
9	tibetanmuseum.org	1	1	1	0.00	1	46	46	46	0.00	46
10	peec.org	10	10	10	0.00	10	2223	2223	2223	0.00	2135
11	americantowns.com/ny~	1	1	1	0.00	1	100	100	100	0.00	98
12	sipcw.org	4	4	4	0.00	4	573	573	573	0.00	552
13	waterfrontalliance.org	10	10	10	0.00	10	5853	5852	5844	0.15	4425
14	hudsonriver.org	3	3	3	0.00	3	320	320	320	0.00	245
15	goingcoastal.org	1	1	1	0.00	1	1	1	1	0.00	1
16	nyopenwater.org	2	2	2	0.00	2	159	160	160	0.63	159
17	596acres.org	10	10	10	0.00	10	952	952	952	0.00	920
18	farmschoolnyc.org	3	3	3	0.00	3	125	125	125	0.00	124
19	rpa.org	4	4	4	0.00	4	2205	2202	2208	0.27	2054
20	sites.google.com/site~	3	3	3	0.00	3	305	305	308	0.97	317
p1	scgis.org/chapters/pages~	1	1	1	0.00	1	53	53	53	0.00	53
p2	americanscottishfoundation.org	1	1	1	0.00	1	35	35	35	0.00	34
p3	biobus.org	1	1	1	0.00	1	23	23	23	0.00	25
p4	cresli.org	1	1	1	0.00	1	83	83	83	0.00	83
p5	littleneck.net/lionsclub~	1	1	1	0.00	1	3	3	3	0.00	3
p6	sinorthshoreresilience.org	1	1	1	0.00	1	43	43	43	0.00	43
mean					0.00					1.48	
median					0.00					0.00	

Table S6. Reasons why 11 sites had a maximum search depth of one. Six cases were valid instances of a maximum depth of one and were retained in the final data set (n = 78). Five cases had larger depths in reality and the snaWeb package was unable to scrape these revealing limitations or sensitivities. We excluded these five cases from our data set (see column "kept").

Site URL	Reason	Details	Kept
americanscottishfoundation.org	Redirect	Home page redirects to a sub-page of the root so no subsequent sub-pages are recognized as sub-pages	no
biobus.org	Redirect	Home page redirects to a sub-page of the root so no subsequent sub-pages are recognized as sub-pages	no
cresli.org	Redirect & Query string	Home page redirects to a sub-page of the root so no subsequent sub-pages are recognized as sub-pages; and page coding uses .cfm and all sub-pages contain a query string (i.e., "?")	no
sinorthshoreresilience.org	Query string	Sub-pages present, but use a query string (i.e., "?")	no
americantowns.com/ny/statenisland/ organization/friends-of-abandoned- cemeteries-inc-s-i	Sub-page logic	Search URL is a sub-page and there are no sub-pages of the URL string in the page's source code.	yes
littleneck.net/lionsclub/index.htm	Sub-page logic	Search URL is a sub-page and there are no sub-pages of the URL string in the page's source code.	yes
nycgovparks.org/trees/ tree-care/ny-tree-trust	Sub-page logic	Search URL is a sub-page and there are no sub-pages of the URL string in the page's source code.	yes
scgis.org/chapters/pages/ new-york-and-new-jersey	Sub-page logic	Search URL is a sub-page and there are no sub-pages of the URL string in the page's source code.	yes
ssa.ccny.cuny.edu/programs-centers/ graduate-programs/m-land-arch-i/	Sub-page logic	Search URL is a sub-page and there are not sub-pages of the URL string in the page's source code.	yes
goingcoastal.org	No link in source code	There are no internal or external hyperlink in the source code, even though the website has a header with sub-page links. This content does not appear in the source code.	yes
tibetanmuseum.org	406 error	Sub-pages are present and picked up by the snaBrowser tool, but all have status "406 error message," which indicates incompatibility between the client request and server response.	no

S.5. Complete List of scraped URLs

The following is the complete list of final 78 scraped URLs:

1 www.596acres.org
2 www.americantowns.com/ny/statenisland/organization/friends-of-abandoned-cemeteries-inc-s-i
3 www.AmpleHarvest.org
4 www.ampletableforeveryone.org
5 www.anhd.org
6 www.aslany.org
7 www.beaconcchc.com
8 www.billionoysterproject.org

9 www.bioboat.org
10 www.carnegiehillcsa.org
11 www.ccmworldwide.com
12 www.centralamericanlegal.info
13 www.CurtisHS.org
14 www.cuspproject.org
15 www.elcentronyc.org
16 www.empirecleancities.org
17 www.fabscrap.org
18 www.farmingconcrete.org
19 www.farmschoolnyc.org
20 www.fowcas.org
21 www.ghanaiancivicassoc.wordpress.com
22 www.goingcoastal.org
23 www.gothamwhale.org
24 www.greenmap.org/nyc
25 www.hudsonriver.org
26 www.hudsonsailing.org
27 www.intervine.nyc
28 www.kayakstatenisland.org
29 www.Lighthousemuseum.org
30 www.lincnyc.org
31 www.littleneck.net/lionsclub/index.htm
32 www.makerspace.nyc
33 www.naturalareasnyc.org
34 www.nature.org/en-us/about-us/where-we-work/united-states/new-york
35 www.NewYorkMYC.org
36 www.nocdny.org
37 www.ny4p.org
38 www.nyc.cce.cornell.edu
39 www.nycgovparks.org/trees/tree-care/ny-tree-trust
40 www.nych2o.org
41 www.nycid.org
42 www.nylcv.org
43 www.nynjbaykeeper.org
44 www.nyopenwater.org
45 www.nysunworks.org
46 www.peec.org
47 www.pluspool.org
48 www.rebuildnyc.org
49 www.rootsandshoots.org
50 www.rpa.org
51 www.scgis.org/chapters/pages/new-york-and-new-jersey
52 www.shorewalkers.org
53 www.sichamber.com
54 www.sigardenclubs.org
55 www.sijcc.org
56 www.sipcw.org

57 www.siprotectors.org
58 www.sisbdc.org
59 www.sites.google.com/site/cubscoutpack37statenisland
60 www.ssa.ccny.cuny.edu/programs-centers/graduate-programs/m-land-arch-i
61 www.sscolumbia.org
62 www.statenislandoutloud.org
63 www.statenislandpps.org
64 www.stgeorgetheatre.com
65 www.streetlab.org/uni-project
66 www.swimmablenyc.org
67 www.taikoza.com
68 www.thePOOPproject.org
69 www.threelakescouncil.org
70 www.tottenvillehistory.com
71 www.troop5527.blogspot.com
72 www.troutintheclassroom.org
73 www.urbanjustice.org
74 www.usi.nyc
75 www.waterfrontalliance.org
76 www.welcometocup.org
77 www.whyhunger.org
78 www.wisonline.org
